# Progress in Surgery

**Published:** 1897-06-19

**Authors:** 


### PROGRESS IN SURGERY.

### GENITO-URINARY SURGERY.

**Vesical Calculus.**—Drs. Laurie and Leon have published[1] a case in which a vesical calculus was clearly seen in a skiagram of the pelvis. The patient was a boy aged eight years. They also experimented with calculi outside the body, and with a calculus placed in the kidney in the cadaver. A skiagram taken of calculi outside the body showed that those composed of oxalate or phosphate of lime were more opaque than bone, uric acid calculi were of almost the same opacity, and gall stones very slightly more opaque than flesh. The calculus (which was of uric acid) placed in the kidney of the cadaver showed as a faint but distinct shadow in the skiagram. Drs. Swain and Cotton have also made experiments with calculi,[2] and find that oxalate of lime gives the densest shadow, phosphatic calculi a shadow decidedly less dense, and uric acid calculi one of greatly diminished density. They have not found the density of shadow always in proportion to the specific gravity of the stone. Uric acid calculi of a greater specific gravity than phosphatic calculi gave a shadow of less density. It is thought that the presence of lime salts always makes the shadow deep. Attention is called to the fact that the shadow caused by a uric acid calculus is less deep than that given by a piece of rib, though the calculus was thicker, and much less dense than that given by a rib, and portion of kidney superimposed. It is suggested that, therefore, the diagnosis of uric acid calculus in the kidney is likely to be a matter of some difficulty.

Surgeon-Lieutenant-Colonel Keegan[3] strongly advocates litholapaxy in the treatment of vesical calculus in boys. He says surgeons in India are more and more adopting this method in preference to lateral lithotomy, and he shows from statistics that the mortality is less in litholapaxy than in lateral lithotomy in boys. He considers the operation so much to be preferred to lateral lithotomy for boys about seven or eight years of age, that the latter should not be performed, but that all cases of stone in boys of this age should be dealt with by experienced lithotritists. The operation in India, though performed with such marked success, seems in many cases to have been undertaken by those not specially experienced in litholapaxy. Suprapubic lithotomy has only very rarely been performed on boys in India, and Keegan thinks the lateral operation much better in cases in which lithotomy is required for some special reason. Keegan also shows[4] how rare calculus in the bladder is in women as compared with men—only 94 out of 3,757 cases of vesical calculus in India were in women. This would show that the disease is about forty times more common in men than in women. He considers that litholapaxy should be the treatment to be almost invariably adopted for vesical calculus in women. Out of the 94 cases 88 were treated by litholapaxy, and only one died. Dilatation of the urethra for extraction of the stone he considers quite unjustifiable, except for the very smallest possible calculi. If lithotomy has to be performed he is inclined to think that vaginal is preferable to suprapubic in these days of aseptic surgery. He thinks that the clean incision in the vaginal and bladder walls is readily stitched together, and if the vagina be rendered aseptic by filling it with iodoform gauze, and a soft catheter be tied in the bladder, perfect union should take place in a few days.

Suprapubic lithotomy is very seldom performed in India, even in cases of large stones in adults. Indeed, Keegan goes so far as to say that experience shows that suprapubic lithotomy is not more successful than lateral lithotomy in dealing with very large calculi, and advocates perineal lithotrity in preference to it. He says that expert litholapaxists in India now attack a stone weighing between two and four ounces with the utmost confidence. He shows from statistics that the marked success of the Indian litholapaxist is not due to an imagined greater tolerance of the natives of India for vesical operations, but to the skill of the operator, acquired by the frequent opportunities which exist for performing the operation. Indian surgeons, who now have so small a mortality in so enormous a number of cases, started with just the same kind of patients and diseases that they now operate on, but had a very much higher mortality at first.

Mr. Henry Morris[6] is not inclined to adopt suprapubic lithotomy as the best method of treating stones which for special reasons cannot be removed by litholapaxy, but prefers perineal lithotrity, even in cases of enlarged prostate, the pelvis in such cases being well raised by placing a firm pillow under the buttocks, or by placing the patient in the Trendelenberg position. He records a case in which he performed perineal lithotrity for calculus formed around a piece of catheter, in an old man, the subject of stricture. Death occurred, probably, from chronic surgical kidneys.

Tilden Brown, of New York, considers[7] the advisability of distending the bladder with air instead of fluid in suprapubic cystotomy. By means of circular letters he has ascertained the practice of surgeons in New York and two other large American cities, and finds that very few use air inflation, a good many use Trendelenberg's position together with injection of the bladder with fluid, and a fairly large proportion still use the rectal bag. The distention of the bladder with air instead of fluid was first proposed by Bristow in 1893.[8] He considers that air, being an extremely elastic and compressible gas, would be less likely to cause rupture of a diseased bladder than fluid; and that it lifts the bladder out of the pelvis and brings it into contact with the anterior abdominal wall, and lifts up the peritoneum much better than fluid does, the weight of the water keeping the bladder low down in the pelvis. Another advantage of air is, that when the bladder is opened there is no flooding of the wound with fluid. Tilden Brown uses a bicycle air-pump with a special chamber attached, holding cotton-wool, which acts as a filter for the air. He says if the bladder is distended with air both the Petersen rectal bag and the Trendelenberg posture can be dispensed with. He exposes the transversalis fassia before inflating the bladder. Before the operation is commenced, the bladder is first washed out with an antiseptic solution. Keen[9] used inflation of the bladder with air as a method of determining rupture as long ago as 1870. If the bladder is not ruptured a resonance hypogastric swelling forms; if it is, the peritoneal cavity becomes exceedingly tympanitic.

[1] Lancet, Jan. 16, 1897. [2] Bristol Med. Chirurg. Journal, March, 1897, p. 1. [3] Lancet, Jan. 16, p. 165. [4] Ibid., Jan. 9, p. 92. [5] Ibid., Jan. 30, p. 299. [6] Middlesex Hospital Journal, Jan. 1897, p. 8. [7] Annals of Surgery, Feb., 1897, p. 141. [8] Annals of Surgery, June, 1893. [9] Annals of Surgery, July, 1890, p. 35.

### SURGERY OF THE PERITONEUM, &c.

**The Absorption of Fluids by the Peritoneum** has been the subject of experiments by Robinson.[1] He regards it as more of a physical than a vital process. It cannot be due to vital-cell processes, for absortion proceeds many hours after death, and, moreover, such absorption is not interfered with by energetic chemical or thermic injuries to the peritoneal endothelium. Intra-abdominal pressure is, on the other hand, a distinct factor in peritoneal absorption. Intra-abdominal pressure may be regarded as muscular tension plus atmospheric pressure, and up to a certain point the amount of absorption is in direct ratio to the amount of pressure. Intra-abdominal pressure and filtration are closely associated, and the latter process is distinctly helpful. Capillary or molecular inhibition is difficult to dispose of, but the probabilities are in favour of its playing a considerable *rôle* in the process. Osmosis and the presence of stomata are also beneficial, but the author does not think that peritoneal fluids are directly absorbed by the blood vessels. Peritoneal absorption must therefore be sought along the lines of osmosis, the inter-endothelial stomata, and filtration.

**Peritoneal Infection** is discussed by Durham,[2] who points out that a considerable degree of resistance against pathogenic microbes is afforded by inducing leucocytosis. He therefore suggests intraperitoneal injections of anti-streptococcic serum the day before operation on "dangerous" cases through the peritoneum, as by this means a specific passive immunity would be afforded against streptococcal invasion, as well as increased general resistance against other microbes. The protection is due to two factors: 1. Increased bactericidal power of the peritoneal fluids after injection. 2. The presence of a very large number of phagocytes, which are not seen in normal peritoneal fluid.[3]

**Tuberculous Peritonitis.**—Gatti[4] thinks that laparotomy has little effect when the peritonitis is quite initial. Cure occurs through a degeneration of the epitheloid cells, without the intervention of wandering cells, independently of phagocytosis, and without the formation of fresh connective tissue. The serous fluid which is thrown out the first few days stimulates the repressive process after laparotomy; this is effected by the serous fluids bathing the tuberculous mass, and having a bactericidal and attenuating action on the tubercle bacilli. D'Urso,[5] however, affirms that cure takes place by leucocytic invasion, organisation of fresh connective tissue, vascular new formation, and substitution of tuberculous tissue] by inflammatory neoformations. Washing out the peritoneal cavity does not act in virtue of any particular antiseptic or anti-tuberculous property of the solution, but mechanically by setting up a certain amount of inflammation, and the washing assists this partly through the disturbance which the sponging out of the fluid demands.

**The Sub-Peritoneal Tissue.**—The diseases of this structure are thus classified by Anderson[6]: I. Inflammations: (A) Classified according to etiology — (1) Idiopathic, (2) tuberculous, (3) syphilitic, (4) metastatic, (5) traumatic, (6) consecutive (parietal, peritoneal, visceral, lymphatic, or remote origin). (B) Classified according to locality—(1) Retroperitoneal (including mesocolic, mesenteric, &c.), (2) subphrenic, (3) perinephric, (4) iliac, (5) pelvic (including parametric), (6) prevesical. II. Neoplasms: (A) Primary—(1) Lipoma and fibro-lioma; (*a*) retroperitoneal (may become mesenteric, mesocolic, omental, or parametric); (*b*) hernial, through inguinal, femoral, or obturator openings, through the linea alba, and in other situations; (*c*) intraperitoneal, after the manner of appendices epiploicæ; (2) fibroma, (3) myoma and fibromyoma, (4) sarcoma, (5) cysts, simple, hydatid, dermoid, &c. (B) Secondary—Carcinoma or sarcoma conveyed by lymphatics or blood-vessels. III. Hæmorrhages: From rupture of healthy or diseased vessels. IV. Lymph exudations: From injury or disease of the receptaculum chyli or its tributaries. V. Extravasation of secretions or excretions: Bile, fæces, &c. VI. Gaseous infiltrations: Air from communication with lung, intestinal gases from perforation of gut, gases of decomposition. VII. Atrophy: Leading to displacement of organs, as in movable kidney, hernia, &c.

**Drainage in Abdominal Surgery** is, according to Bishop,[7] distinctly required in all operations on tubes, wherever practicable, and in all septic operations; is beneficial in cases where pure asepticity is doubtful, and is distinctly harmful in cases which are aseptic, and in which the patency of a tube is not involved.

**The Intraperitoneal Treatment of Abdominal Hydatids** is advocated by Russel and Ryan.[8] Russel points out that there is absolutely no reason for treating the sac in the ordinary way, by suturing it to the abdominal wall. For all practical purposes the sac may be regarded as only the capsule which necessarily forms round a foreign body, and when the foreign body which has excited its formation has been removed the adventitious sac may be disregarded and dropped back into the depths of the wound, and the wound closed completely, as after any ordinary abdominal operation.

**Gunshot Wounds of Abdomen.**—Parker[9] advises early and rapid operation. Diagnosis is generally easy, but when there is doubt he would advise enlarging the wound or probing. In doubtful cases of injury of the intestine he is inclined to attach much importance to pain referred to the umbilicus. As to the technique of operation: (1) Unless the wound is well to one side a median incision is best. (2) All the intestine should be examined. (3) Peritoneal wounds are best united by Lembert's suture. (4) In wounds of the liver packing is better than suturing. (5) Drainage, except in late cases, is not necessary if all hæmorrhage has been stopped. (6) If the intestine cannot be sutured without great risk of obstruction, resection should be performed. (7) Unless the bullet can be felt search should not be made for it.

**Fæcal Tumour.**—Gersuny[10] describes a sign which, if detected, will save a patient from needless laparotomy. If the tumour be firmly pressed on with the finger the intestinal mucous membrane adheres to the sticky masses of which it is composed; when the pressure is removed the mucous membrane slowly disengages itself, and the sign consists in the recognition of this gradual separation. The only conditions necessary are a certain degree of dryness of the intestinal mucous membrane and a certain impressibility of the fæcal mass.

[1] Annals of Surgery, March, 1897. [2] Brit. Med. Journ., March 13th, 1897, p. 655. [3] Lancet, March 13th, 1897, p. 737. [4] New York Med. Journ., Oct. 10th, 1896. [5] Internat. Med. Mag., Sept., 1896, p. 517. [6] Brit. Med. Journ., Oct. 17th, 1896, p. 1,087. [7] Therapeut. Gazette. Feb. 15th, 1897, p. 124. [8] Med. Rec. New York, Feb. 27th, 1897, p. 303. [9] Ibidem, Dec. 19th, 1896, p. 901. [10] Brit. Med. Journ., Jan. 2nd, 1897.

